# Facile Synthesis of Mg-MOF-74 Thin Films for Enhanced CO_2_ Detection

**DOI:** 10.3390/nano15201541

**Published:** 2025-10-10

**Authors:** Yujing Zhang, Evan J. Haning, Hao Sun, Tzer-Rurng Su, Alan X. Wang, Ki-Joong Kim, Paul R. Ohodnicki, Chih-Hung Chang

**Affiliations:** 1 School of Chemical, Biological, and Environmental Engineering, Oregon State University, Corvallis, OR 97331, USA; zhangyuj@oregonstate.edu (Y.Z.); sutze@oregonstate.edu (T.-R.S.); 2 School of Electrical Engineering and Computer Science, Oregon State University, Corvallis, OR 97331, USA; 3 National Energy Technology Lab, United States Department of Energy, Pittsburgh, PA 15236, USA; ki-joong.kim@netl.doe.gov (K.-J.K.); pro8@pitt.edu (P.R.O.)

**Keywords:** metal–organic frameworks, rapid thin-film synthesis, CO_2_ adsorption, infrared gas sensing

## Abstract

Metal–organic frameworks (MOFs) are a class of highly ordered nanoporous crystals that possess a designable framework and unique chemical versatility. MOF thin films are ideal for nanotechnology-enabling applications, such as optoelectronics, catalytic coatings, and sensing. Mg-MOF-74 has been drawing increasing attention due to its remarkable CO_2_ uptake capacity among MOFs and other commonly used CO_2_ absorbents. Mg-MOF-74 thin films are currently fabricated by immersing selected substrates in precursor solutions, followed by a traditional solvothermal synthesis process. Herein, we introduce a rapid, easy, and cost-effective synthesis protocol to fabricate MOF thin films in an additive manner. In this work, the controllable synthesis of Mg-MOF-74 thin films directly on optical supports is reported for the first time. Dense, continuous, and uniform Mg-MOF-74 thin films are successfully fabricated on bare glass slides, with an average growth rate of up to 85.3 nm min^−1^. The structural and optical properties of the resulting Mg-MOF-74 thin films are characterized using X-ray diffraction, atomic force microscopy, scanning electron microscopy, UV-Vis-NIR spectroscopy, and Fourier Transform Infrared Spectroscopy (FTIR). The CO_2_ adsorption performance of the resulting Mg-MOF-74 thin films is studied using FTIR for the first time, which demonstrates that, as per the length of the light path for gas absorption, 1 nm Mg-MOF-74 thin film could provide 400.9 ± 18.0 nm absorption length for CO_2_, which is achieved via the extraordinary CO_2_ adsorption by Mg-MOF-74. The synthesis protocol enables the rapid synthesis of MOF thin films, highlighting Mg-MOF-74 in more CO_2_-related applications, such as enhanced CO_2_ adsorption and MOF-enhanced infrared gas sensing.

## 1. Introduction

Metal–organic frameworks (MOFs) are highly ordered nanoporous crystals with a designable structure that feature extensive internal porosity and versatile functionality, contributing to the selective adsorption of various contents (e.g., gases, vapors, ions, small chemical compounds) [1]. Their unique porous properties, with a high surface area, demonstrate incomparable adsorption behavior, and their high-yield, low-cost capability makes MOFs promising candidates for clean energy use [2]. Over 20,000 distinct kinds of MOFs have been reported and investigated in the past decade, demonstrating outstanding potential in various applications [1,3,4,5]. 

Within MOFs, the M-MOF-74 family (M = Mg, Zn, Ni, or Co) has attracted increasing attention due to its exceptionally high CO_2_ adsorption capacity compared with other MOFs and conventional CO_2_ absorbents [6,7,8]. M-MOF-74, also known as IRMOF-74-M or CPO-27-M, consists of linear infinite-rod secondary building units (SBUs, i.e., metal ions/clusters) connected by 2,5-dihydroxyterephthalate (denoted as DOBDC) linkers, resulting in a 1D hexagonal porous structure with pore diameter ~12 Å [9]. The combination of pore size, unique topology, and a high density of coordinatively unsaturated metal sites makes M-MOF-74 particularly favorable for CO_2_ adsorption. These unsaturated metal sites are created during synthesis through partial replacement of DOBDC by solvent molecules (typically H_2_O or DMF), which rearranges the coordination environment of metal centers and facilitates strong interactions with CO_2_ [10]. Among the M-MOF-74 series, Mg-MOF-74 has been reported to have an excess uptake capability compared to Ni, Co, and Zn, as indicated by CO_2_ isotherm analysis [11]. Additionally, Mg-MOF-74 has been claimed to perform more than twice the volumetric uptake under atmospheric pressure, which is attributed to the ionic character of the Mg-O bonding [12].

Due to the increased demand for applying MOF in thin-film devices such as sensors, microelectronics, optical electronics, solar cells, and catalytic surfaces, the development of facile thin-film synthesis strategies is necessary. Conventional solvothermal synthesis is known to be a time-consuming process. Additionally, it tends to result in homogeneous precipitation [13], which leads to the formation of free Mg-MOF-74 crystals in the liquid phase, rather than a heterogeneous reaction that promotes thin-film formation. Currently, there is only a handful of reports [8,14,15,16,17,18,19] that explore the synthesis of Mg-MOF-74 thin films. In 2010, Bétard et al. [14] introduced a three-step heating process into the thin film procedure, which originated from the conventional powder synthesis developed by Caskey et al. [11]. Mg-MOF-74 thin films were successfully synthesized on porous alumina substrates with film thickness ranging from 1 to 10 μm under different reaction conditions. However, the heating process involves a first heating step of 20 h, followed by 4 h of slow cooling, and finally, reheating for another 20 h to complete the film formation process. Moreover, the film uniformity was not desirable due to the large size of the crystal formation. A few years later, Wang et al. [15] conducted studies on seeding layers to enhance the heterogeneous nucleation and growth of Mg-MOF-74 thin films via a solvothermal reaction. They discovered that a MgO seeding layer can accelerate the nucleation and growth rates of Mg-MOF-74 thin films on porous alumina substrates. The film formation improvement was suggested by the high dissolution rate of MgO under the synthesis conditions, which releases a large amount of Mg^2+^ ions to react with deprotonated DOBDC linkers in the close vicinity of the substrate. Yet, obtaining a 10 μm Mg-MOF-74 film on MgO-seeded support still required 24 h of synthesis.

Several factors in MOF synthesis critically influence their structural morphology, adsorption capability, and corresponding surface area. Mg-MOF-74 exhibits similar dependencies even in thin film development. Campbell et al. [8] successfully deposited Mg-MOF-74 thin film without seeding support and investigated the influence of reaction time, precursor dose, and solvent composition in the synthesis recipe. The authors suggested that the solvent composition significantly impacts the resulting crystal size and film thickness, which is likely due to the varied solubility of H_4_DOBDC in different solvents. The rapid dissolution rate of the linker, resulting from the high solubility of H_4_DOBDC in DMF, promoted the formation of small crystals (<1 μm in diameter). The increase in dissolution rate indirectly accelerated the crystal nucleation process, which in turn hindered the formation of a uniform thin film. As a result, by replacing a portion of solvent use to water and ethanol mixture in DMF, Mg-MOF-74 films on porous membranes with thicknesses ranging from 1 to 14 µm could be synthesized within 2.5–6 h. In short, although synthesis conditions have been optimized over the years to improve film formation in the Mg-MOF-74 field, no research has achieved a significant reduction in synthesis time. On the other hand, this also highlights the limitations of current optimization strategies. MOF synthesis optimization involves multiple interdependent parameters. Such computational screening of synthesis conditions could provide mechanistic insights into metal-linker coordination and guide the rational design of synthesis protocols to achieve desired morphology [20]. This integrated computational-experimental approach for MOF thin films remains unexplored and represents a promising direction for future research.

Additionally, the substrate surface presents another challenge in MOF thin film deposition processes. Surface roughness of the substrate is a critical factor that significantly influences the quality and performance characteristics of the deposited film [21]. Based on our literature review, most Mg-MOF-74 thin film studies have employed porous alumina as substrates [8,14,15,16,17] or a platinum-patterned silicon wafer [18]. These substrates often provide enhanced adhesion of film coatings by increasing the contact surface area and even offering additional mechanical interlocking due to their uneven surface. Nevertheless, the applications of these Mg-MOF-74 thin films have been confined to gas separation/adsorption, batteries, and capacitive gas sensors.

In this work, we demonstrated a facile synthesis protocol for fabricating Mg-MOF-74 thin film onto glass substrates. The film thickness can be controlled through the number of synthesis cycles, with a high film growth rate. We systematically investigated the effects of precursor concentration and ramping temperature on film properties, as discussed in the section on synthesis modification. The thin film thickness and surface roughness were characterized using SEM and AFM. The successful deposition on a transparent glass substrate enabled the utilization of FTIR to evaluate CO_2_ adsorption through changes in infrared absorption. Consequently, the synthesized Mg-MOF-74 films demonstrate significantly enhanced CO_2_ adsorption performance, with an average effective absorption length of 400.9 ± 18.0 nm per nanometer of film thickness.

## 2. Materials and Methods

Materials and Chemicals. Micro Slides (25 × 75 × 1.0 mm^3^) were purchased from VWR International. Magnesium acetate tetrahydrate (Mg(CH_3_COO)_2_·4H_2_O, 98%, Sigma-Aldrich, Burlington, MA, USA), 2,5-dihydroxyterephthalic acid (H_4_DOBDC, 98%, TCI, Portland, OR, USA), dimethylformamide (DMF, >99.8%, Sigma-Aldrich), anhydrous methanol (>99.8%, Macron Fine Chemicals, Swedesboro, NJ, USA), ethanol (>99.5%, Sigma-Aldrich), sulfuric acid (H_2_SO_4_, 95–98%, J.T. Baker, Radnor, PA, USA), ammonium hydroxide (NH_3_, 28%, Alfa Aesar, Ward Hill, MA, USA), and hydrogen peroxide (H_2_O_2_, 30%, Macron, Phillipsburg, NJ, USA) were used as purchased without further purification. Ar (99.99+%) and CO_2_ (99.99+%) gas tanks were purchased from Airgas.

Preparation of Substrates. One piece of VWR Micro Slides was cut into three pieces, each with a dimension of 25 × 25 × 1.0 mm^3^. The glass slides were first cleaned with soap and deionized water (DIW) and then immersed in piranha solution (containing H_2_SO_4_ and H_2_O_2_ in a volume ratio of 5:3) at room temperature to remove surface contaminants. After 30 min of immersion, the slides were removed from the piranha solution, thoroughly rinsed with DIW, and placed into a new solution containing NH_3_, H_2_O_2,_ and DIW in a volume ratio of 1:1:5, which was subjected to ultrasonication at 70 °C for 1 h to increase the hydrophilicity of the substrate to improve film formation. The slides were then thoroughly rinsed with DIW and stored in a batch of fresh DIW before experiments.

Fabrication of Mg-MOF-74 Thin Films. 0.35 mol L^−1^ (M) Mg(CH_3_COO)_2_ solution was prepared with a solvent mixture containing 1.20 mL DMF, 0.40 mL DIW, and 0.40 mL ethanol; while 0.20 M H_4_DOBDC solution was prepared with 2.00 mL DMF. After complete dissolution, the clear brown H_4_DOBDC solution was added to the Mg(CH_3_COO)_2_ solution, followed by agitation to ensure thorough mixing. This mixture solution remained clear and brown, serving as the precursor solution for synthesizing Mg-MOF-74 thin films.

**Scheme 1:**
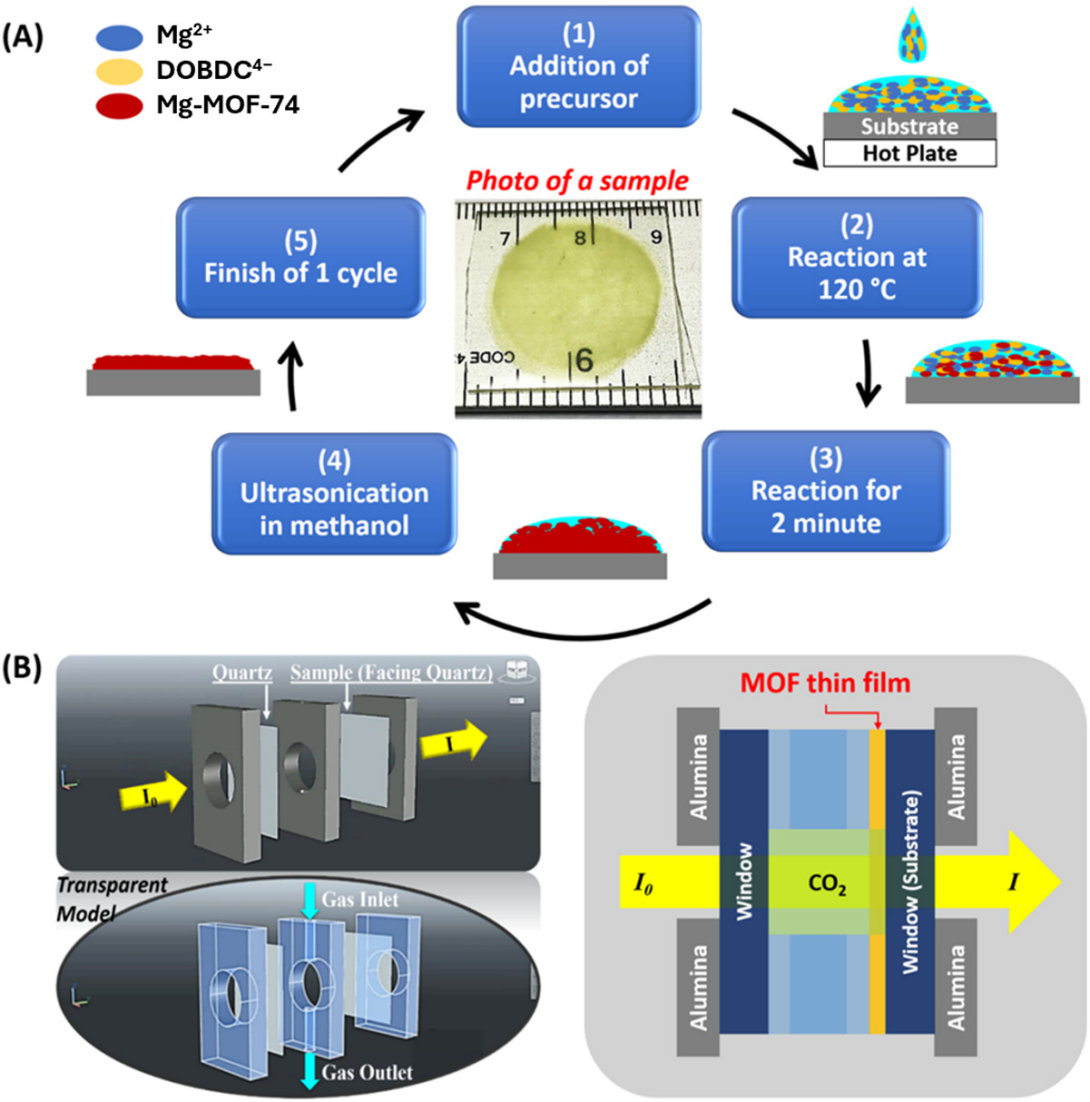
(**A**) Schematics of the rapid synthesis scheme for Mg-MOF-74 thin films. (**B**) Model illustration of the lab-made FTIR gas cell and its working mechanism in the study of gas adsorption in MOF thin films.

A schematic diagram of the synthesis protocol for Mg-MOF-74 thin films is shown in Scheme 1A. A prepared glass substrate was dried with pressurized airflow and placed on a hotplate for 1 min to allow the surface temperature to stabilize at the reaction temperature (T_r_), which was 120 °C. 0.40 mL precursor solution was added onto the substrate and allowed to heat for 2 min. The sample was then removed from the heat, allowed to reach room temperature, and submerged in a beaker containing fresh methanol. While submerged, the beaker was placed under ultrasonication for a minimum of 15 s. The sample was then removed from the beaker and gently rinsed with fresh methanol. If significantly raised particulates remained, ultrasonication was continued for a few additional seconds. Ultrasonication up to 40 s was tested, and no exfoliation of films was observed on any sample. The sample was then dried with pressurized airflow. At this point, one standard synthesis cycle was completed. The process was repeated until the desired layers had been deposited. Samples are named 2L, 3L, 4L, and 6L, respectively, depending on the number of deposited layers. After the deposition was complete, the thin-film samples were submerged under fresh methanol. The methanol bath for the thin films was replaced three times over a period of two days to facilitate solvent exchange. All samples were then activated at 120 °C in a vacuum before characterization.

A temperature-ramping process was introduced into the synthesis protocol to investigate the film formation by allowing additional reaction time. Two alternative reaction schemes were investigated. In the first scheme, we set the substrate surface temperature at an initial temperature (T_i_) of 100 °C. Following the standard additive synthesis procedure, the temperature then increased to 120 °C after staying at 100 °C for 2 min. The sample was then removed from the hotplate, followed by a standard washing process. In the second scheme, the experimental conditions and synthesis procedure remained the same, except that T_i_ was maintained at 80 °C. Likewise, in the following experiments, different combinations of T_r_ and T_i_ were investigated. The resulting films were analyzed.

Additional experiments were conducted at increased organic linker concentrations, paired with a temperature-ramping process, to determine the effect of linker concentration on film formation. By keeping other experimental conditions and synthesis procedure constant, we used different linker concentrations (0.24, 0.28, and 0.30 M) in the synthesis of Mg-MOF-74 thin films. The resulting film morphology was analyzed.

Study of Gas Adsorption. The CO_2_ adsorption property of Mg-MOF-74 was studied via Fourier-transform infrared spectroscopy (FTIR). A customized FTIR gas cell (Scheme 1B) was built to regulate the samples in the study of gas adsorption. The cell, constructed from three plates with circular holes in the center, allows light to pass through. A channel is made in the middle plate to direct gas flow. The central plate and two windows, which comprise a quartz slide and a glass substrate with a thin-film sample grown on it, define the gas cell, measuring 7 mm in diameter × 4 mm in length. The side of the glass substrate with the Mg-MOF-74 thin film faces the center of the gas cell. Mass flow controllers were used for switching gas flows between Ar and CO_2_. Ar gas was used to purge the system, and a blank glass window was studied with CO_2_ to establish a baseline reading. Then, Mg-MOF-74 thin films with varying thickness were analyzed for CO_2_ adsorption.

Characterization. The crystal structure of the resulting thin films was determined using a Bruker D8 Discover X-ray diffraction (XRD) system, operating at 40 kV and 40 mA with Cu Kα radiation of 1.5405 Å, in the 2θ scan range from 5° to 20° with a step size of 0.05°. A reference diffraction pattern for Mg-MOF-74 was simulated via Materials Studio. The morphology of Mg-MOF-74 thin films was first analyzed by a Bruker Veeco Innova scanning probe microscope with the tapping mode atomic force microscope (AFM), and then by an FEI Quanta 600F Environmental Scanning Electron Microscope (SEM) under high vacuum with a gold coating of about 8 nm in thickness on top of the sample surface. An acceleration voltage of 15.00 kV and a spot size of 2.5 were applied. Film thickness was estimated based on the cross-sectional images of each sample. The absorption spectra of Mg-MOF-74 thin films deposited on glass substrates were collected in attenuated total reflection (ATR) mode of a Thermo Scientific Nicolet 6700 Fourier transform infrared (FTIR) spectrometer.

## 3. Results and Discussion

**Figure 1:**
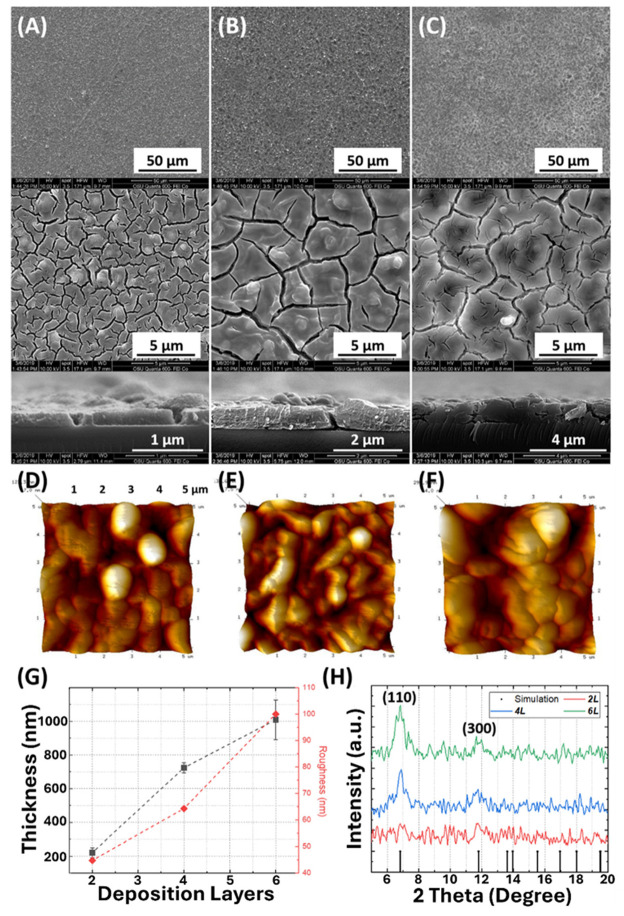
(**A**–**C**) Top-view and cross-section SEM and (**D**–**F**) top-view AFM images of 2L, 4L, and 6L, respectively. (**G**) Average film thickness and roughness as a function of deposition layers. (**H**) XRD patterns of 2L, 4L, 6L, and a simulated reference.

The Mg-MOF-74 film quality was evaluated using top-view and cross-sectional imaging via SEM (Figure 1A–C). The results show that all the Mg-MOF-74 thin films are continuously grown on the substrates with relatively high uniformity. However, several cracks and poorly intergrown particles can be observed under higher magnification. In contrast, the cracks are absent in the AFM images (Figure 1D–F), which further suggests that the defects observed in the SEM image originated from the intense e-beam. These film damages can also be observed in other reported MOF films [22,23]. As shown in Figure 1G, a linear relationship exists between the number of deposition cycles (2L–6L) and the overall film thickness. Each deposition cycle results in a layer with an average thickness of 153.4 ± 30.6 nm under the reaction conditions employed (a metal/organic precursor concentration ratio of 3:2 and no temperature-ramping process, with T_i_ = T_r_ = 120 °C). By comparing the XRD patterns of 2L, 4L, and 6L with the simulated diffraction pattern of Mg-MOF-74 (Figure 1H), it is confirmed that Mg-MOF-74 thin films showing two preferred growth directions are successfully synthesized via the rapid thin-film synthesis protocol. The diffraction peaks at 2θ of approximately 6.8° and 11.7° are attributed to the (110) and (300) planes of Mg-MOF-74, respectively. While XRD analysis provides a good agreement of successful synthesis, we noticed that the intensity is lower compared to other studies. This reduced intensity can be attributed to (1) a relatively high noise-to-signal ratio due to the glass slide substrate, which typically produces higher background signals during XRD analysis, and (2) lower crystallinity compared to film from hours-long reactions. On the other hand, the low crystallinity is reflected in the Mg-MOF-74 crystals observed in SEM images. The images reveal a predominantly spherical morphology of individual crystals across all samples. As the reaction occurs within a short timeframe, structural morphology evolution is not observed, even with the modified recipe described in a later section. Nevertheless, the resulting film can be effectively created with controllable thickness using our rapid method (2 min per deposition cycle).

**Figure 2:**
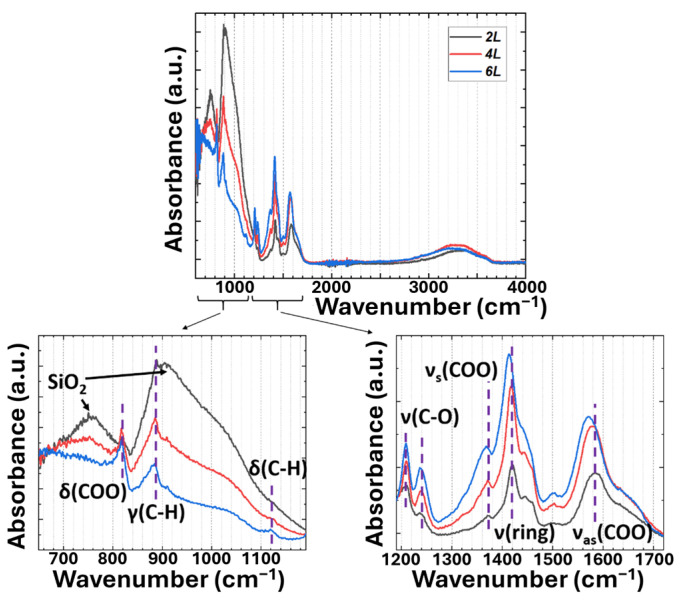
FTIR ATR spectra of 2L, 4L, and 6L, respectively. Note: stretching (ν), bending in-plane (δ), and out-of-plane modes (γ).

According to the FTIR absorption spectra in Figure 2, all the IR peaks corresponding to the linker exhibit a redshift with increasing the deposition layers, which could result from the formed conjugated system and the increased mass of Mg-MOF-74 [24]. Moreover, the absorption from SiO_2_ is reduced upon an increase in the deposition layers, indicating an increased thickness of Mg-MOF-74 on the substrate. It suggests that Mg-MOF-74 does not exhibit any absorption in the short-wavelength IR region, which corresponds to a few characteristic adsorption bands of CO_2_ around 2300 and 3700 cm^−1^, thereby enabling it to be utilized as a spectroscopic indicator for investigating CO_2_ adsorption in Mg-MOF-74.

**Figure 3:**
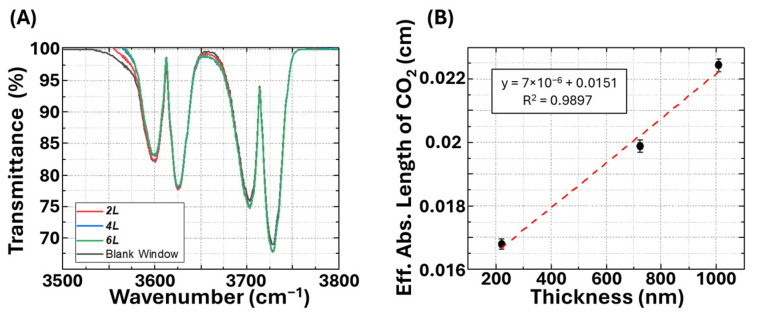
(**A**) The as-collected FTIR transmission spectra of 99.99+% CO_2_ with different window sets in the gas cell. (**B**) Extra absorption length of 99.99+% CO_2_ as a function of the thickness of Mg-MOF-74 thin films.

The experimental design for gas adsorption was based on previous investigations [25,26,27,28]. We studied the gas adsorption properties of resulting Mg-MOF-74 thin films with varying thickness using FTIR in transmission mode, as illustrated in Scheme 1B. The transmittance data obtained from 2L, 4L, 6L, and the blank window are shown in Figure 3A. To evaluate the CO_2_ adsorption enhancement by the Mg-MOF-74 thin film, baseline spectra were first obtained using a blank glass substrate (without Mg-MOF-74) as one gas cell window, which served as the reference for all subsequent measurements. The absorbance originates solely from CO_2_ in the gas cell, and the absorption coefficient (α) of CO_2_ can be calculated via the Beer-Lambert law relation (Equation (1)).

(1) $$\frac{I_{out}}{I_{in}}=e^{\left(-\alpha L\right)}$$

where L is the 4 mm gas cell path length, *I_in_* and *I_out_* are the incident and transmitted IR intensities from the FTIR, and α is the absorption coefficient (cm^−1^). Once the Mg-MOF-74 thin film is introduced, CO_2_ molecules are adsorbed within the porous medium, resulting in stronger adsorption than the gas cell alone. The overall absorption can then be expressed as Equation (2).

(2) $$\frac{I_{out}}{I_{in}}=e^{\left[-\alpha \left(L+L^{\prime }\right)\right]}$$

where L′ is the effective absorption length associated with uptake in the Mg-MOF-74 film. Through this calculation, the influence of the intrinsic absorption from both the substrate and the MOF thin film can be eliminated, leaving only the IR absorption of CO_2_ for investigation. The enhanced IR absorption of CO_2_ is presented as the effective absorption length of CO_2_ in the Mg-MOF-74 thin film (after subtracting the 4 mm light path in the original gas cell) and is shown in Figure 3B. The calculated absorption was plotted, which reveals that different thicknesses of Mg-MOF-74 lead to varied effective absorption lengths of CO_2_. Moreover, a linear relationship is established, where the enhanced CO_2_ IR adsorption intensity increases proportionally with the thickness of the Mg-MOF-74 thin film. The results showed that a 1 nm Mg-MOF-74 thin film could provide an absorption length of 400.9 ± 18.0 nm for CO_2_. This achieved a 100-fold absorption enhancement per nanometer of Mg-MOF-74 compared to the blank device, which was attributed to the exceptional adsorptive behavior. This result demonstrates the excellent CO_2_ adsorption capability of Mg-MOF-74, and the enhanced CO_2_ adsorption via this gas concentrating effect is vital for CO_2_ IR sensing.

Modification of Synthesis Procedure. To investigate the influence of reaction conditions on the formation of Mg-MOF-74 thin films and identify optimal fabrication conditions, several controlled experiments were conducted by varying key synthesis parameters, including reaction time, temperature, heating process, and precursor dosage. The following paragraphs will discuss these influencing parameters and their effects on film formation.

**Figure 4:**
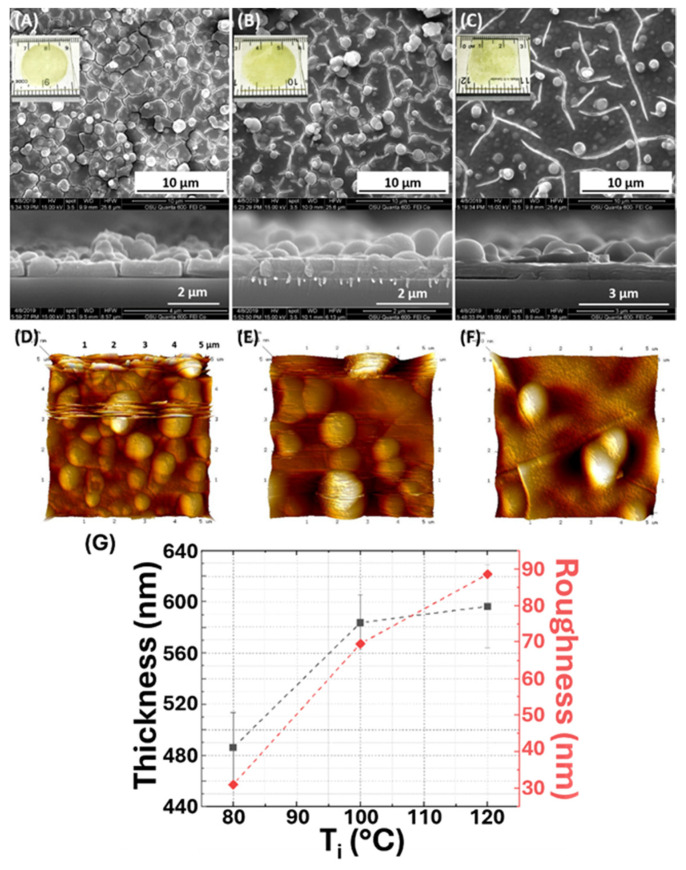
(**A**–**C**) Top-view and cross-section SEM and (**D**–**F**) top-view AFM images of 3L samples synthesized at different T_i_. (**A**) No temperature ramping as a control, T_i_ = T_r_ = 120 °C, (**B**) T_i_ = 100 °C and T_r_ = 120 °C, and (**C**) T_i_ = 80 °C and T_r_ = 120 °C. The images inserted into the SEM images are the optical photos of the corresponding samples, deposited on 1-inch × 1-inch glass substrates. (**G**) Average film thickness and roughness as a function of T_i_.

By fixing other reaction conditions, a temperature-ramping process was introduced into the synthesis procedure. Temperature ramping can substantially influence the reaction temperature and extend the reaction time by slowing down the solvent evaporation rate. Compared with preliminary studies that did not include a temperature-ramping process, allowing approximately two minutes for reaction in each cycle before the solvent was completely dried, the temperature-ramping process extended the reaction time to about four and a half minutes by adding a 2 min ramping step. Based on observations via SEM and AFM (Figure 4A–F), the synthesis of T_r_, Mg-MOF-74 thin films with different T_i_ doses reveals a varied surface morphology. When decreasing T_i_, the resulting thin film appears denser under the intense electron beam from the SEM, as evidenced by fewer cracks. Moreover, a less spherical crystal structure is formed at a decreased T_i_ concentration, indicating a better intergrowth for the thin film. In addition, the Mg-MOF-74 film thickness decreased as the lower initial temperature was used (Figure 4G). Lowering the T_i_ concentration reduces the solvent evaporation rate, substantially extending the overall reaction time and resulting in improved intergrowth for the thin-film structure. However, lower temperatures also reduce the growth rate, resulting in a thinner film during formation. Therefore, optimal temperature-ramping conditions should be carefully selected to balance the good intergrowth of the thin film with achieving the desired thickness.

**Figure 5:**
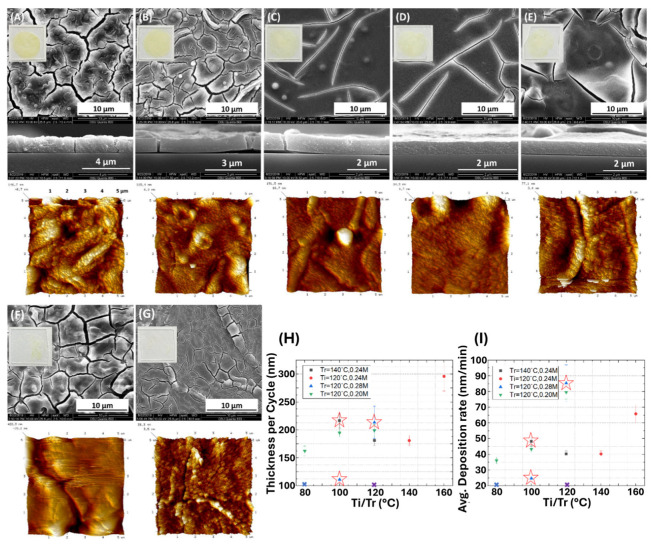
Top-view and cross-section SEM and AFM images of 4L samples synthesized under different reaction conditions. Concentration of organic linker: (**A**–**C**) 0.24, (**D**–**F**) 0.28, and (**G**) 0.30 M. Temperature-ramping process: (**A**) T_i_ = 120 °C and T_r_ = 160 °C, (**B**) T_i_ = 120 °C and T_r_ = 140 °C, (**C**) T_i_ = 100 °C and T_r_ = 140 °C, (**D**) T_i_ = 100 °C and T_r_ = 120 °C, (**E**) T_i_ = T_r_ = 120 °C, (**F**) T_i_ = 80 °C and T_r_ = 120 °C, and (**G**) T_i_ = T_r_ = 120 °C. The images inserted in the SEM are optical photos of the corresponding samples deposited on glass substrates (1 inch × 1 inch). (**H**) Average thin film thickness per deposition cycle, and (**I**) growth rate (thickness per minute of reaction) under different temperature-ramping processes and different doses of precursors, in which the hollow stars indicate the best-intergrown films (**C**–**E**).

When fixing the concentration of metal precursors, the influence of organic linker concentration on the formation of Mg-MOF-74 thin film was studied. Similar to the previous investigation on the reaction temperature, the resulting thin films exhibit a surface morphology difference when the precursor dosage is changed, as shown in Figure 5A–G. The concentration of the organic linker plays a crucial role in controlling the intergrowth behavior of the MOF thin film by influencing the nucleation of MOF formation [29]. A high concentration of organic linkers can induce a rapid nucleation rate, thereby producing more secondary building units or nano-sized crystals that can eventually benefit the intergrowth. According to the SEM and AFM images in Figure 5E,G, increasing the concentration of the organic linker reasonably improved the uniformity and intergrowth of the thin film, eliminating the need for a temperature-ramping process. Excessive linker concentrations would result in defective film formation rather than well-grown thin films. When the concentration of the organic linker reaches 0.30 M, or when it is 0.28 M at a low temperature (T_i_ = 80 °C), the resulting film exhibits weaker adhesion and tends to exfoliate after ultrasonication. The film thickness of each sample was estimated using the cross-sectional SEM images, except for the samples shown in Figure 5F,G, due to the exfoliation of the films. The summarized plot of film thickness prepared under different conditions is shown in Figure 5H, along with the corresponding growth rate under each reaction condition in Figure 5I. When fixing T_i_ and T_r_, increasing the organic linker concentration from 0.20 to 0.28 M enhanced the growth rate from 72.3 to 85.3 nm/min. Moreover, the results indicate that the reaction temperature could significantly affect the film growth rate. The growth rate increases from 40.2 to 65.7 nm min^−1^ when T_i_ is fixed and T_r_ is increased from 140 to 160 °C. In contrast, the growth rate increases from 43.2 to 79.5 nm min^−1^ when fixing T_r_ and increasing T_i_ from 80 to 120 °C. Therefore, it suggests that the organic linker concentration has a substantial impact on the nucleation rate, which in turn influences the degree of intergrowth in the thin film and the resulting uniformity. At the same time, the reaction temperature affects the crystal growth rate, which alters the resulting thickness of the Mg-MOF-74 thin film. Hence, the application of an optimal organic linker concentration and the temperature-ramping process can result in Mg-MOF-74 thin films of the desired quality. This work primarily focuses on the synthesis conditions and factors that affect the formation of Mg-MOF-74 thin films via this rapid synthesis method. However, our optimized reaction conditions enable the successful formation and present the fastest growth rate of 85.3 nm min^−1^ without the need for seeding support. Indeed, its trade-off behavior via this film deposition method needs to be further investigated.

## 4. Conclusions

The rapid synthesis of continuous Mg-MOF-74 thin films was successfully demonstrated on glass slides using a controllable and time-saving strategy, eliminating the need for self-assembled monolayers or seeding layers. The film thickness can be easily adjusted by changing the deposition layers of the precursors. By applying a temperature-ramping step and modifying the dose of precursors, Mg-MOF-74 thin films with uniform thickness and good integrity were obtained under the reaction conditions employed. Under our optimized reaction conditions, an average growth rate of 85.3 nm min^−1^ is achieved, surpassing the documented fastest growth rate for Mg-MOF-74 thin films on smooth surfaces, which is approximately 14.2 nm min^−1^ [18]. The newly developed protocol enables the fabrication of Mg-MOF-74 thin films on smooth, unseeded optical supports for the first time. Meanwhile, this cost-effective method not only reduces reaction time compared to traditional synthesis but also offers excellent budget affordability, significantly broadening the opportunity for Mg-MOF-74 to a wide range of applications.

Excellent CO_2_ adsorption in Mg-MOF-74 was confirmed via FTIR, and the corresponding results indicated that a 1 nm Mg-MOF-74 thin film could provide an average effective absorption length for CO_2_ of 400.9 ± 18.0 nm, attributed to the gas concentrating effect. The realization of rapid synthesis of Mg-MOF-74 thin films on glass slides via a simple and effective additive method not only contributes to the development of MOF thin-film synthesis, but also showcases a promising morphology for Mg-MOF-74 in various applications in the research field of adsorption/separation [30,31,32,33], catalysis [34], proton transportation [35], drug delivery [36], and enhanced IR gas sensing.

## Data Availability

Data available on request from the authors.

## References

[1] Furukawa H., Cordova K.E., O’Keeffe M., Yaghi O.M. (2013). The chemistry and applications of metal-organic frameworks. Science.

[2] Li S.-L., Xu Q. (2013). Metal–organic frameworks as platforms for clean energy. Energy Environ. Sci..

[3] Shekhah O., Liu J., Fischer R., Wöll C. (2011). MOF thin films: Existing and future applications. Chem. Soc. Rev..

[4] Sharmin E., Zafar F. (2016). Introductory chapter: Metal organic frameworks (MOFs). Metal-Organic Frameworks.

[5] Wang H., Zhu Q.-L., Zou R., Xu Q. (2017). Metal-organic frameworks for energy applications. Chem.

[6] Krishna R., van Baten J.M. (2011). In silico screening of metal–organic frameworks in separation applications. Phys. Chem. Chem. Phys..

[7] Krishna R., van Baten J.M. (2012). A comparison of the CO_2_ capture characteristics of zeolites and metal–organic frameworks. Sep. Purif. Technol..

[8] Campbell J., Tokay B. (2017). Controlling the size and shape of Mg-MOF-74 crystals to optimise film synthesis on alumina substrates. Microporous Mesoporous Mater..

[9] Cadot S., Veyre L., Luneau D., Farrusseng D., Quadrelli E.A. (2014). A water-based and high space-time yield synthetic route to MOF Ni 2 (dhtp) and its linker 2, 5-dihydroxyterephthalic acid. J. Mater. Chem. A.

[10] Dietzel P.D., Johnsen R.E., Blom R., Fjellvåg H. (2008). Structural changes and coordinatively unsaturated metal atoms on dehydration of honeycomb analogous microporous metal–organic frameworks. Chem.–A Eur. J..

[11] Caskey S.R., Wong-Foy A.G., Matzger A.J. (2008). Dramatic tuning of carbon dioxide uptake via metal substitution in a coordination polymer with cylindrical pores. J. Am. Chem. Soc..

[12] Lackner K.S., Wendt C.H., Butt D.P., Joyce Jr E.L., Sharp D.H. (1995). Carbon dioxide disposal in carbonate minerals. Energy.

[13] Ameloot R., Vermoortele F., Vanhove W., Roeffaers M.B., Sels B.F., De Vos D.E. (2011). Interfacial synthesis of hollow metal–organic framework capsules demonstrating selective permeability. Nat. Chem..

[14] Bétard A., Zacher D., Fischer R.A. (2010). Dense and homogeneous coatings of CPO-27-M type metal–organic frameworks on alumina substrates. CrystEngComm.

[15] Wang N., Mundstock A., Liu Y., Huang A., Caro J. (2015). Amine-modified Mg-MOF-74/CPO-27-Mg membrane with enhanced H2/CO_2_ separation. Chem. Eng. Sci..

[16] Gholidoust A., Maina J.W., Merenda A., Schütz J.A., Kong L., Hashisho Z., Dumee L.F. (2019). CO_2_ sponge from plasma enhanced seeded growth of metal organic frameworks across carbon nanotube bucky-papers. Sep. Purif. Technol..

[17] Luo J., Li Y., Zhang H., Wang A., Lo W.-S., Dong Q., Wong N., Povinelli C., Shao Y., Chereddy S. (2019). Metal-Organic Framework Thin Film for Selective Mg^2+^ Transport. Angew. Chem. Int. Ed..

[18] Yuan H., Tao J., Li N., Karmakar A., Tang C., Cai H., Pennycook S.J., Singh N., Zhao D. (2019). On-Chip Tailorability of Capacitive Gas Sensors Integrated with Metal–Organic Framework Films. Angew. Chem. Int. Ed..

[19] Kim K.-J., Culp J.T., Ohodnicki P.R., Thallapally P.K., Tao J. (2021). Synthesis of High-Quality Mg-MOF-74 Thin Films via Vapor-Assisted Crystallization. ACS Appl. Mater. Interfaces.

[20] Pela R.R., Hsiao C.-L., Hultman L., Birch J., Gueorguiev G.K. (2024). Electronic and optical properties of core-shell InAlN nanorods: A comparative study via LDA, LDA-1/2, mBJ, HSE06, G_0_W_0_ and BSE methods. Phys. Chem. Chem. Phys..

[21] Liu J., Wöll C. (2017). Surface-supported metal-organic framework thin films: Fabrication methods, applications, and challenges. Chem. Soc. Rev.

[22] Hou C., Xu Q., Peng J., Ji Z., Hu X. (2013). (110)-Oriented ZIF-8 Thin Films on ITO with Controllable Thickness. ChemPhysChem.

[23] Henkelis S.E., Percival S.J., Small L.J., Rademacher D.X., Nenoff T.M. (2021). Continuous MOF Membrane-Based Sensors via Functionalization of Interdigitated Electrodes. Membranes.

[24] Karmakar B. (2016). Fundamentals of glass and glass nanocomposites. Glass Nanocomposites.

[25] Chong X., Kim K.-j., Zhang Y., Li E., Ohodnicki P.R., Chang C.-H., Wang A.X. (2017). Plasmonic nanopatch array with integrated metal–organic framework for enhanced infrared absorption gas sensing. Nanotechnology.

[26] Chong X.Y., Zhang Y.J., Li E.W., Kim K.J., Ohodnicki P.R., Chang C.H., Wang A.X. (2018). Surface-Enhanced Infrared Absorption: Pushing the Frontier for On-Chip Gas Sensing. ACS Sens..

[27] Chong X., Kim K.-J., Li E., Zhang Y., Ohodnicki P.R., Chang C.-H., Wang A.X. (2016). Near-infrared absorption gas sensing with metal-organic framework on optical fibers. Sens. Actuators B Chem..

[28] Zhang Y., Chong X., Sun H., Kedir M., Kim K.-J., Ohodnicki P., Wang A., Chang C.-H. (2020). Nanostructured CuS Thin Film via a Spatial Successive Ionic Layer Adsorption and Reaction Process Showing Significant Surface-Enhanced Infrared Absorption of CO_2_. J. Mater. Chem. C.

[29] Öztürk Z., Filez M., Weckhuysen B.M. (2017). Decoding Nucleation and Growth of Zeolitic Imidazolate Framework Thin Films with Atomic Force Microscopy and Vibrational Spectroscopy. Chem.–A Eur. J..

[30] Perry I.V., J J., Teich-McGoldrick S.L., Meek S.T., Greathouse J.A., Haranczyk M., Allendorf M.D. (2014). Noble gas adsorption in metal–organic frameworks containing open metal sites. J. Phys. Chem. C.

[31] Böhme U., Barth B., Paula C., Kuhnt A., Schwieger W., Mundstock A., Caro J., Hartmann M. (2013). Ethene/ethane and propene/propane separation via the olefin and paraffin selective metal–organic framework adsorbents CPO-27 and ZIF-8. Langmuir.

[32] Bao Z., Alnemrat S., Yu L., Vasiliev I., Ren Q., Lu X., Deng S. (2011). Adsorption of ethane, ethylene, propane, and propylene on a magnesium-based metal–organic framework. Langmuir.

[33] Su X., Bromberg L., Martis V., Simeon F., Huq A., Hatton T.A. (2017). Postsynthetic functionalization of Mg-MOF-74 with tetraethylenepentamine: Structural characterization and enhanced CO_2_ adsorption. ACS Appl. Mater. Interfaces.

[34] Yao H.-F., Yang Y., Liu H., Xi F.-G., Gao E.-Q. (2014). CPO-27-M as heterogeneous catalysts for aldehyde cyanosilylation and styrene oxidation. J. Mol. Catal. A Chem..

[35] Solís C., Palaci D., Llabres i Xamena F.X., Serra J.M. (2014). Proton Transport through Robust CPO-27-type Metal Organic Frameworks. J. Phys. Chem. C.

[36] Erucar I., Keskin S. (2017). Computational investigation of metal organic frameworks for storage and delivery of anticancer drugs. J. Mater. Chem. B.

